# Comparative Effects of Teriparatide and Risedronate in Glucocorticoid-Induced Osteoporosis in Men: 18-Month Results of the EuroGIOPs Trial

**DOI:** 10.1002/jbmr.1870

**Published:** 2013-01-15

**Authors:** Claus-C Glüer, Fernando Marin, Johann D Ringe, Federico Hawkins, Rüdiger Möricke, Nikolaos Papaioannu, Parvis Farahmand, Salvatore Minisola, Guillermo Martínez, Joan M Nolla, Christopher Niedhart, Nuria Guañabens, Ranuccio Nuti, Emilio Martín-Mola, Friederike Thomasius, Georgios Kapetanos, Jaime Peña, Christian Graeff, Helmut Petto, Beatriz Sanz, Andreas Reisinger, Philippe K Zysset

**Affiliations:** 1 Sektion Biomedizinische Bildgebung, Klinik für Diagnostische Radiologie, Universitätsklinikum Schleswig-HolsteinKiel, Germany; 2 Lilly Research CenterWindlesham, UK; 3 Klinikum LeverkusenLeverkusen, Germany; 4 Hospital 12 de OctubreMadrid, Spain; 5 Institut für Präventive Medizin & Klinische ForschungMagdeburg, Germany; 6 Laboratory for the Research of Musculoskeletal System, University of Athens Medical School, KAT HospitalAthens, Greece; 7 Policlinico Umberto IRoma, Italy; 8 Hospital BellvitgeBarcelona, Spain; 9 GemeinschaftspraxisHeinsberg, Germany; 10 Hospital Clinic and Center of Network Biomedical Research in Hepatic and Digestive DiseasesBarcelona, Spain; 11 Policlinico Le ScotteSiena, Italy; 12 Hospital La PazMadrid, Spain; 13 Osteoporose StudiengesellschaftFrankfurt, Germany; 14 Aristotelion UniversityThessaloniki, Greece; 15 Lilly Research CenterVienna, Austria; 16 Institute for Lightweight Design and Structural BiomechanicsVienna, Austria; 17 Institute of Surgical Technology and BiomechanicsBern, Switzerland

**Keywords:** BONE MINERAL DENSITY, BONE MICROSTRUCTURE, FINITE ELEMENT ANALYSIS, GLUCOCORTICOID-INDUCED OSTEOPOROSIS, HIGH RESOLUTION QUANTITATIVE COMPUTED TOMOGRAPHY, MALE OSTEOPOROSIS, VERTEBRAL FRACTURE

## Abstract

Data on treatment of glucocorticoid-induced osteoporosis (GIO) in men are scarce. We performed a randomized, open-label trial in men who have taken glucocorticoids (GC) for ≥3 months, and had an areal bone mineral density (aBMD) *T*-score ≤ –1.5 standard deviations. Subjects received 20 μg/d teriparatide (*n* = 45) or 35 mg/week risedronate (*n* = 47) for 18 months. Primary objective was to compare lumbar spine (L_1_–L_3_) BMD measured by quantitative computed tomography (QCT). Secondary outcomes included BMD and microstructure measured by high-resolution QCT (HRQCT) at the 12th thoracic vertebra, biomechanical effects for axial compression, anterior bending, and axial torsion evaluated by finite element (FE) analysis from HRQCT data, aBMD by dual X-ray absorptiometry, biochemical markers, and safety. Computed tomography scans were performed at 0, 6, and 18 months. A mixed model repeated measures analysis was performed to compare changes from baseline between groups. Mean age was 56.3 years. Median GC dose and duration were 8.8 mg/d and 6.4 years, respectively; 39.1% of subjects had a prevalent fracture, and 32.6% received prior bisphosphonate treatment. At 18 months, trabecular BMD had significantly increased for both treatments, with significantly greater increases with teriparatide (16.3% versus 3.8%; *p* = 0.004). HRQCT trabecular and cortical variables significantly increased for both treatments with significantly larger improvements for teriparatide for integral and trabecular BMD and bone surface to volume ratio (BS/BV) as a microstructural measure. Vertebral strength increases at 18 months were significant in both groups (teriparatide: 26.0% to 34.0%; risedronate: 4.2% to 6.7%), with significantly higher increases in the teriparatide group for all loading modes (0.005 < *p* < 0.015). Adverse events were similar between groups. None of the patients on teriparatide but five (10.6%) on risedronate developed new clinical fractures (*p* = 0.056). In conclusion, in this 18-month trial in men with GIO, teriparatide showed larger improvements in spinal BMD, microstructure, and FE-derived strength than risedronate.

## Introduction

Glucocorticoids (GCs) are widely prescribed for the treatment of inflammatory, autoimmune, and allergic disorders. It has been estimated that approximately 0.2% to 0.5% of the general population is receiving GCs.^1^ However, their chronic use is the most common cause of secondary osteoporosis. A consequence of glucocorticoid-induced osteoporosis (GIO) is bone fragility and an increased risk for low-trauma fractures^2^ that are frequently the presenting manifestations of this disorder. Fractures have been reported in 30% to 50% of patients receiving long-term GC therapy.^3^,^4^ Importantly, the fracture risk was shown to be higher in patients with GIO than in patients with postmenopausal osteoporosis for the same level of areal bone mineral density (aBMD)^5^ measured by dual X-ray absorptiometry (DXA), probably because of the additional effects of muscle weakness and frailty, and changes in bone material properties that are not captured by aBMD. The classical measurement of aBMD by DXA has the disadvantage of being based on a two-dimensional (2D) image of a three-dimensional (3D) structure, which reveals no information on the depth of the bone and does not distinguish trabecular and cortical bone. Thus, the reported improvements in aBMD with osteoporosis therapies, although clinically useful, do not necessarily reflect an accurate estimate of restored volumetric bone mineral density (BMD) or bone strength in patients with GIO.

Due to GCs' primary effect of a profound inhibition of osteoblastic bone forming activity, a need exists for therapies that can substantially improve bone formation and the microarchitecture status of patients with GIO. Along with such therapies, more sensitive diagnostic and evaluation methods beyond DXA, such as quantitative computed tomography (QCT) or assessment of cancellous microstructure with either high-resolution QCT (HRQCT)^6^–^8^ or magnetic resonance imaging (MRI) techniques should be applied.^9^ Moreover, vertebral bone strength as the most important determinant of fracture risk can be assessed in vivo by a simulated mechanical test based on the BMD distribution using finite element (FE) analysis.^10^ Both QCT and HRQCT permit the separate measurement of trabecular and cortical BMD, the latter offering more accurate results on cortical and trabecular microstructure but at the expense of a higher radiation dose (1–2 mSv for QCT of L_1_–L_3_, and 2–3 mSv for HRQCT of T_12_). QCT has long been used to assess vertebral fracture risk, to measure age-related bone loss, and in follow-up of osteoporosis and other metabolic bone diseases. The validity of this technique for measurement of vertebral cancellous bone is widely accepted.^11^ Evaluations of BMD by QCT or HRQCT have recently been reported in the assessment of the effects of full-length human parathyroid hormone (PTH[1–84]) and teriparatide in postmenopausal women with osteoporosis^7^,^12^–^16^ or with GIO,^17^ as well as in men with osteoporosis.^18^ However, there are no randomized controlled studies on treatment of GIO in men using these technologies. Moreover, the effects of the different therapies for GIO in men have been poorly studied.^19^

The primary objective of this study was to test the hypothesis that teriparatide, a bone-forming drug, was superior to risedronate, a pyridinyl bisphosphonate that reduces bone turnover, in improving lumbar spine BMD measured by QCT over 18 months in males with GIO. Furthermore, we compared the treatment effects of both drugs using recently developed imaging techniques, such as BMD and microstructure measured by HRQCT, and vertebral body strength computed by HRQCT-based nonlinear FE analysis.

## Patients and Methods

### Study design

Patients at 16 centers in Germany, Greece, Italy, and Spain were enrolled in this phase 3, randomized, open-label, active comparator controlled study, which was conducted between July 2007 and October 2010. The study consisted of two study periods: a screening phase of up to 6 weeks, and an 18-month open-label treatment phase. At the baseline visit, patients were randomized in a 1:1 ratio to either 20 µg teriparatide once a day as a subcutaneous injection or 35 mg risedronate once weekly orally as tablet. Randomization was done centrally and stratified by previous bisphosphonate use; ie, whether the patient used bisphosphonates for a total of at least 1 month at any time prior to study entry. Any previous osteoporosis treatment given at the time of screening was discontinued before randomization and for the duration of the study. After randomization, patients received the study medication for 18 months, with clinical visits occurring after 3, 6, 12, and 18 months. All patients were to concomitantly receive 1 g elemental calcium and 800 to 1200 IU of vitamin D per day during the study.

The study was approved by the responsible institutional review boards at each center, and was conducted in accordance with the ethical principles of the Declaration of Helsinki, good clinical practices, and applicable laws and regulations. The patients' written informed consent had been obtained before conducting any study procedures.

### Participants

Male ambulatory outpatients aged ≥25 years with normal laboratory values for serum calcium, alkaline phosphatase, 25-hydroxyvitamin D, and PTH were enrolled. They had a lumbar spine (L_1_–L_4_), femoral neck, or total hip BMD *T*-score of at least 1.5 SDs below the corresponding normal young adult man average BMD. For inclusion, at least two lumbar vertebrae were required to be without imaging artifacts, fractures, or other abnormalities that would interfere with the DXA and QCT assessments. Subjects had to have received GC therapy at an average dose of at least 5.0 mg/d of prednisone or its equivalent for a minimum of 3 consecutive months immediately preceding the screening visit. Exclusion criteria included presence of skeletal diseases other than GIO, prevalent spinal fractures at both the 12th thoracic vertebra (T_12_) and L_1_, impaired renal function (creatinine clearance <30 mL/min), abnormal thyroid function not corrected by therapy, history of symptomatic nephrolithiasis or urolithiasis in the year prior to randomization, malignant neoplasms in the 5 years prior to randomization, and any contraindication to therapy with teriparatide or risedronate. Patients were also excluded if they had taken intravenous bisphosphonates within 12 months prior to the screening visit, or strontium ranelate or fluoride at therapeutic doses (≥20 mg/d) for more than 3 months in the 2 years prior to randomization, or for more than a total of 2 years, or at any dose within the 6 months prior to randomization. Previous treatment for any duration with calcitonin, oral bisphosphonates, or active vitamin D3 analogues that had been stopped prior to or at the randomization visit was allowed.

### Efficacy measures

QCT and HRQCT scans were obtained at months 0, 6, and 18. The primary endpoint in the EuroGIOPs trial was the change in trabecular BMD (Tb.BMD) of L_1_–L_3_ at month18; its measurement at 6 months was a secondary endpoint. Further secondary efficacy endpoints included HRQCT variables, strength (failure load) and stiffness at T_12_ as estimated by FE analysis, aBMD of the lumbar spine, total hip, and femoral neck measured by DXA, and biochemical markers of bone turnover including serum amino-terminal propeptide of type I procollagen (P1NP) and serum beta C-terminal cross-linking telopeptide of type I collagen (β-CTx).

### Assessment methods for efficacy measures

The efficacy endpoints of this trial were evaluated by trained experts at a central imaging laboratory in Kiel, Germany, using validated image analysis software for QCT and HRQCT; by trained experts at an engineering laboratory in Vienna, Austria, for FE analysis; by a central reading center for DXA analysis and quality assurance (BioImaging Technologies, Leiden, The Netherlands); and by a central laboratory for serum analytics (Covance, Geneva, Switzerland). All central facilities, as well as the personnel at the local radiology departments of the study sites, were blinded to treatment assignment.

#### QCT imaging and analysis

**Fig. 1:**
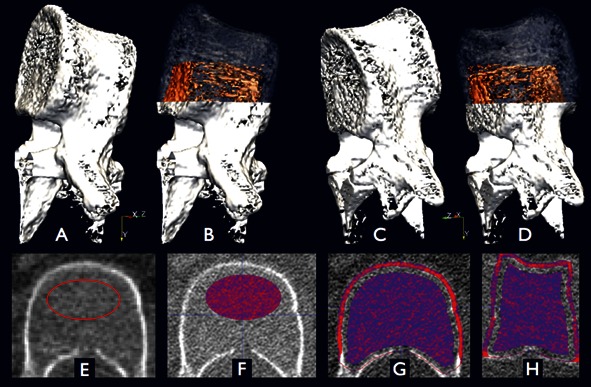
2D and 3D visualization of a vertebra imaged noninvasively by high-resolution quantitative computed tomography. The 3D images of the top row show a vertebra viewed from two angles differing by 180 degrees (*A*, *B* versus *C*, *D*). The semitransparent versions (*B*, *D*) depict the cortical endplates and elements of the trabecular microstructure of a selected subvolume (colored brown within the semitransparent vertebral body). The bottom row shows placement of the volume of interest (VOI) within the vertebral body in the axial (*E*–*G*) and sagittal (*H*) planes. For HRQCT (*F*–*H*) a cortical region (endplates and lateral and anterior cortices depicted on *G* and *H*) and two different trabecular regions (elliptical in *F* and entire volume in *G* and *H*), except a 2-mm subcortical endosteal region, were evaluated. The elliptical region in *F* was defined similar to the standard QCT region shown, for comparison, on a regular resolution QCT image in *E*. Compared to QCT, HRQCT demonstrates improved delineation of cortical bone (*E* versus *F*).

To assess Tb.BMD, a continuous spiral computed tomography (CT) covering all of L_1_–L_3_ was acquired at 120 kV and 100 mA. The slice thickness was 3 mm; pixel size was in the order of 0.6 mm, but could vary between clinical sites. Quality assurance and BMD calibration were carried out using the dedicated phantoms and procedures provided by Mindways, Inc. (Austin, TX, USA). All scans were centrally evaluated in a volume of interest (VOI) with an elliptical cross-section (Fig. 1*E*) with the software QCTPRO version 4.1.3 (Mindways, Inc.) following the guidelines of the manufacturer. This included calibration and field uniformity correction measures using two phantoms provided by Mindways, Inc. Tb.BMD was calculated for each vertebra and also as an average of all lumbar vertebrae measured. Vertebrae judged as fractured by the central radiologist were not included in the analysis.

#### HRQCT imaging and analysis

For HRQCT, a thin-slice spiral CT scan of T_12_ was acquired at 120 kV and 360 mA. If there was a fracture in T_12_, the HRQCT was performed on an intact L_1_ vertebra. The images were density-calibrated using the same phantoms and methods as for QCT. Technical details of the procedure have recently been published.^20^ The complete vertebral body was segmented using a semiautomatic algorithm of the software tool Structural*Insight*.^7^,^20^ HRQCT permits visualization of the 3D trabecular microstructure, although partial volume effects lead to depiction as a coarser structure (Fig. 1*A*–*D*). Compared to QCT, a sharper delineation of the cortical contours can be achieved (Fig. 1*E* versus *F*), which also permits differentiation of thicker and thinner or porous endplates (Fig. 1, top row). The inner contour of the cortex was defined using a combination of active shape, and global and local thresholding (Fig. 1*G*, *H*). The final contours of the cortex could be modified in 3D by the user if the automated procedure did not produce satisfactory results. Bone density of the bone compartment has been referred to as tissue mineral density (TMD).^21^ Because we cannot resolve cortical pores, TMD will be affected by mineral density of pure bone matrix and porosity. Moreover, because the endosteal cortical-trabecular transition is gradual, the cortical region may include some endosteal marrow space, and thus we conservatively refer to the density in the cortical region as cortical BMD (Ct.BMD), not TMD. As microstructural measure for the cortex, apparent cortical thickness (app.Ct.Th) was assessed directly in 3D using an adaptation of an algorithm reported by Krebs and colleagues.^8^ An additional measure of cortical thickness was derived, this one weighted by local cortical BMD relative to an assumed full mineralization of 1200 mg/cm^3^ to correct for partial volume effects (density weighted cortical thickness [Ct.Th.DW]).

In addition to the cortex, a trabecular region was defined by removing the cortex and the outer 2 mm of the remaining vertebral body to avoid endosteal subcortical bone of higher density (Fig. 1*G*, *H*). A second elliptical trabecular region was evaluated (Fig. 1*F*), very similar to the elliptical region used on QCT images (Fig. 1*E*), and BMD was measured for both trabecular VOIs. For microstructural characterization of the trabecular regions apparent bone volume fraction (app.BV/TV) and apparent trabecular separation (app.Tb.Sp) were assessed by binarization with a fixed, predefined threshold of 250 mg/cm^3^.^7^ App.Tb.Sp was defined as the median length of all sections within the background (marrow) of the search grid of the directed secant method.^22^ This approach was also used to calculate apparent trabecular number (app.Tb.N) as total number of intersection per total test length. App. BV/TV and app.Tb.N were used to derive apparent bone surface-to-volume ratio (app.BS/BV) as 2 × [app.Tb.N]/[app.BV/TV]. Trabecular TMD could be calculated from BMD/[BV/TV] but for the trabecular compartment result will be even more heavily affected by partial volume effects. Therefore, we did not include this in the set of variables to be evaluated statistically. Still, interpretation of relative changes under treatment may provide useful insights and are helpful in the context of the discussion of the results. The prefix “app.” was added to standard nomenclature of structural variables to indicate the influence of limited resolution and signal-to-noise ratio.^7^

#### FE analysis

Digital FE models were generated for each patient and for each HRQCT time point from the segmented HRQCT images at an isometric resolution of 1.3 mm. The superior and inferior endplates were embedded in a virtual thin layer of polymethylmethacrylate and the mineral density of each voxel/element was converted to bone volume fraction (BV/TV) with a calibration equation assuming a homogeneous normal tissue density. The bone tissue material behavior was elasto-plastic with damage; ie, irreversible strain flow and elastic modulus decrease with postyield loading history. The model generation procedure and bone material properties have been described in detail by Chevalier and colleagues.^23^ In order to account for a broad spectrum of physiological loading, FE analysis of each vertebral body included axial compression, anterior bending, and axial torsion.^24^ The structural output variables were stiffness (kN/mm) and maximal load (kN) for axial compression, and angular stiffness (kNmm/rad) and maximal torque (kN/mm) for anterior bending and axial torsion. A normalized strength in axial compression (N/mm^2^ = MPa) was calculated as strength divided by the central cross-sectional area of the vertebral body.^25^

#### DXA

Lumbar spine (L_1_–L_4_), total hip, and femoral neck aBMD were measured by DXA. aBMD results of the total hip obtained on Hologic, GE-Lunar, and Norland scanners were converted to standardized values, and aBMD results of the lumbar spine and femoral neck obtained on Lunar and Norland scanners were converted to Hologic values using published and validated formulas.^26^,^27^ All DXA instruments used in the trial were standardized and cross-calibrated through the use of an anthropomorphic spine phantom (Bona Fide Phantom [BFP], BioImaging Technologies, Leiden, The Netherlands).

#### Biochemical markers of bone turnover

Serum concentrations of P1NP and β-CTx were determined at baseline, 3, 6, and 18 months of treatment. Serum samples were prepared and stored at –20°C or lower at the study site and sent to the central laboratory for processing. All samples from an individual were assayed in a single analytical batch. Serum P1NP was measured by the Intact UniQ RIA assay (Orion Diagnostica, Espoo, Finland). The interassay (within day) analytical coefficient of variation (CV) was 3.1% to 8.2% over the reference interval. Serum β-CTx was measured by the serum Crosslaps Enzyme Linked Immunosorbent Assay (Nordic Bioscience Diagnostics, Herlev, Denmark).The interassay CV was 5.4% to 11.4%.

### Safety measures

Safety measures included pre-existing conditions and treatment-emergent adverse event (TEAEs), physical examination, body weight, height, and body mass index (BMI), vital signs, new clinical fractures, and hypercalcemia defined as a serum calcium level corrected for albumin of >2.7 mmol/L (>10.8 mg/dL).

### Statistical analysis

It was estimated that a sample size of 31 subjects in each group would give at least 85% power to detect a between-treatment difference in the mean change of BMD from baseline to 18 months of 11.25 g/cm^3^, assuming a common SD of 15 g/cm^3^. This was based on a two-sample *t* test at the 5% significance. In the preplanned primary analysis, a mixed-model repeated measures (MMRM) model was evaluated for the change from baseline in lumbar spine trabecular BMD. The same MMRM model was applied to secondary efficacy data.

The prespecified primary MMRM model (“full model”) for change from baseline in the different primary and secondary efficacy endpoints included fixed effects for treatment, visit, interaction between treatment and visit, baseline result of the respective diagnostic variable, age, baseline P1NP, fracture within 12 months prior to study (yes/no), duration of prior bisphosphonate use, baseline GC dose and cumulative GC doses before and during the study, and patient nested within treatment (as random effect). The primary comparison between treatments was assessed at 18 months and, as part of the secondary analysis, at 6 months. Within treatments, the least square (LS) mean changes (given also as percentage changes from baseline) with standard errors and *p* values were analyzed and reported at 6 and 18 months.

To assess the relevance of the baseline adjustments, and as supportive analyses, a “reduced” MMRM model with fixed effects for treatment, visit, the interaction between treatment and visit, and baseline value of the respective diagnostic variable was also applied on the primary and secondary efficacy measures. To maintain the dataset of the full model, patients with missing data on confounders of the full model were excluded in the reduced model. In addition, changes from baseline to endpoint within treatment groups were tested with *t* tests and nonparametric Wilcoxon tests with last observation carried forward (LOCF) applied to patients with missing observations at month 18.

Efficacy analyses were based on the full analysis set (FAS), which included all randomized patients who received at least 1 dose of study medication. Observations included in the MMRM model were limited to those that had nonmissing values for the change in the diagnostic variable studied, and nonmissing values for all of the confounders. To be included in the primary efficacy analysis, FAS patients also needed to have a trabecular BMD measurement by QCT at baseline and at least one postbaseline visit; similarly, a measurement of the secondary endpoints at baseline and at least one postbaseline measurement for the variable assessed were required for inclusion in the secondary efficacy analysis datasets. Confirmatory efficacy analyses were performed using the per-protocol population that excluded any pre-defined major protocol violators. The safety analysis set used for safety analyses included all patients who received study treatment, and patients were analyzed as treated.

Patients with TEAEs and new clinical fractures were compared between treatments using Fisher's exact test. In addition, the number of clinical fractures was compared in a post hoc analysis using a Poisson regression including treatment as independent variable.

All statistical tests were conducted two-sided at the 5% significance level and no multiplicity adjustments were performed for secondary endpoints. Data were analyzed using SAS software version 9.2 (SAS Institute, Inc., Cary, NC).

## Results

**Fig. 2:**
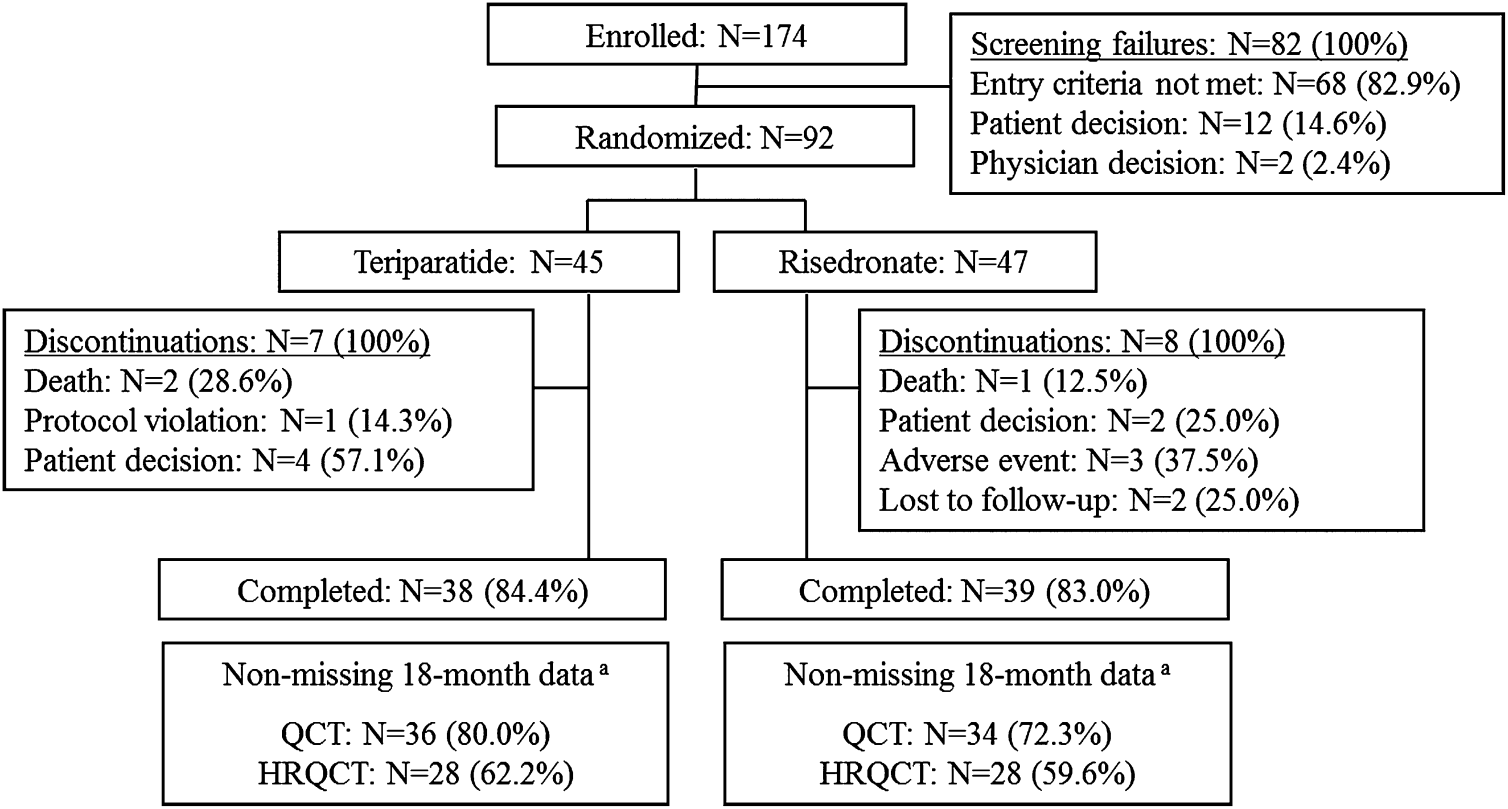
Patient disposition. HRQCT = high resolution quantitative computed tomography; N = total number of patients; QCT = quantitative computed tomography. ^a^Three patients with missing data for covariates included in the full MMRM model (1 teriparatide and 2 risedronate patients).

A total of 174 patients were screened at 19 study sites. Of these patients, 92 were eligible and randomly assigned to teriparatide (45 patients) or risedronate (47 patients) (Fig. 2). A total of 15 patients discontinued the study prematurely, 7 (15.6%) in the teriparatide group and 8 (17.0%) in the risedronate group. Patients' baseline characteristics were generally balanced between treatment groups, with the exception of a higher frequency of anti-tumor necrosis factor (TNF) therapy in the risedronate group (Table 1). Mean age was 56.3 years (range, 25–82 years). In both study groups combined, 36 patients (39.1%) had a previous fracture, and 31 patients (33.7%) had received an osteoporosis therapy prior to the study, mostly bisphosphonates (Table 1). The median GC dose at baseline was 8.8 mg/d, and GCs were mainly taken for rheumatoid arthritis (22.7% of GC requiring disorders), Crohn's disease (14.5%), asthma (10.0%), and chronic obstructive pulmonary disease (8.2%), for a median duration of 6.4 years (Table 2).

**Table 1:** Baseline Characteristics

Variable	Teriparatide	Risedronate	Total
Age (years)			
*n*	45	47	92
Mean (SD)	57.5 (12.8)	55.1 (15.5)	56.3 (14.2)
Race: Caucasian, *n* (%)	44 (97.8)	46 (97.9)	90 (97.8)
Anthropometry			
*n*	45	47	92
Body mass index (kg/m^2^), mean (SD)	27.2 (5.0)	26.5 (4.2)	26.9 (4.6)
Height (cm), mean (SD)	172.2 (7.8)	170.0 (9.7)	171.1 (8.8)
Weight (kg), mean (SD)	80.9 (16.7)	76.8 (14.1)	78.8 (15.5)
Patients with ≥1 previous osteoporosis therapy, *n* (%)	14 (31.1)	17 (36.2)	31 (33.7)
Any bisphosphonate	14 (31.1)	16 (34.0)	30 (32.6)
Duration of prior bisphosphonate use (months)			
*n*	13	16	29
Median (Q1, Q3)	4.0 (2.0, 5.0)	4.5 (2.0, 23.5)	4.0 (2.0, 22.0)
Patients with ≥1 previous anti-TNF therapy, *n* (%)	5 (11.1)	10 (21.3)	15 (16.3)
aBMD (by DXA)			
*n*	45	47	92
Lumbar spine (*T*-score)	–2.48 (1.01)	–2.33 (1.19)	–2.40 (1.11)
Total hip (*T*-score)	–1.64 (0.87)	–1.51 (0.90)	–1.57 (0.88)
Femoral neck (*T*-score)	–1.95 (0.78)	–1.82 (0.91)	–1.88 (0.85)
BMD (by QCT)			
*n*	39	40	79
Lumbar spine L_1_–L_3_ (mg/cm^3^)	75.7 (28.8)	78.2 (29.5)	77.0 (29.0)
Prevalent fractures^a^			
*n*	45	47	92
Subjects with ≥1 fracture(s) prior to study, *n* (%)	19 (42.2)	17 (36.2)	36 (39.1)
Fracture <12 months before study, *n* (%)	4 (8.9)	4 (8.5)	8 (8.7)
Number of fractures per patient^b^			
Mean (SD)	1.8 (0.96)	2.0 (1.22)	1.9 (1.08)
Time since last fracture (months)			
Median (Q1, Q3)	27.8 (15.3, 106.0)	44.9 (10.4, 91.6)	31.5 (10.4, 91.6)
Patients with vertebral fractures, *n* (%)	15 (42.9)	18 (52.9)	33 (47.8)
Patients with nonvertebral fractures, *n* (%)	20 (57.1)	16 (47.1)	36 (52.2)
SDI			
*n*	44	45	89
Mean (SD)	1.7 (2.9)	1.0 (1.5)	1.3 (2.3)
Hormonal and bone markers			
25-Hydroxy vitamin D (pmol/mL)			
*n*	45	46	91
Mean (SD)	64.6 (23.7)	55.0 (29.0)	59.7 (26.8)
Serum PTH (1–84) (pmol/L)			
*n*	45	47	92
Mean (SD)	3.6 (1.07)	3.7 (1.56)	3.6 (1.34)
Total testosterone (ng/dL)			
*n*	32	31	63
Mean (SD)	432.0 (153.8)	416.9 (187.4)	424.5 (169.9)
β-CTx (ng/mL)			
*n*	42	47	89
Mean (SD)	0.4 (0.18)	0.4 (0.22)	0.4 (0.20)
P1NP (µg/L)			
*n*	42	47	89
Mean (SD)	31.7 (22.9)	34.6 (20.1)	33.2 (21.4)

*n* = number of patients with available data; Q1 = lower quartile; Q3 = upper quartile; TNF = tumor necrosis factor; aBMD = areal bone mineral density; DXA = dual-energy X-ray absorptiometry; BMD = bone mineral density; QCT = quantitative computerized tomography; SDI = spinal deformity index; PTH = parathyroid hormone; β-CTx = type I collagen degradation fragments; P1NP = amino-terminal propeptide of type I procollagen.

a Between the age of 21 and study entry.

b Prior to the study, a total of 35 fractures (including 8 [22.9%] due to severe trauma) occurred in the teriparatide group compared to 34 (including 8 [23.9%] due to severe trauma) in the risedronate group.

**Table 2:** Previous Glucocorticoid Use

Variable	Teriparatide	Risedronate	Total
Glucocorticoid dose at baseline (mg/d)			
*n*	44	43	87
Median (Q1, Q3)	8.8 (5.0, 15.0)	8.8 (5.0, 12.5)	8.8 (5.0, 15.0)
Glucocorticoid cumulative dose (g)			
*n*	45	47	92
Median (Q1, Q3)	20.0 (8.3, 43.5)	15.3 (4.6, 32.0)	15.8 (6.3, 37.5)
Duration of prior glucocorticoid treatment (years)			
*n*	45	47	92
Median (Q1, Q3)	7.1 (2.3, 13.2)	4.9 (2.5, 12.9)	6.4 (2.4, 13.0)
Underlying glucocorticoid-requiring disorders, *n* (%)^2^			
Rheumatoid arthritis	11 (19.0)	14 (26.9)	25 (22.7)
Crohn's disease	5 (8.6)	11 (21.2)	16 (14.5)
Asthma	8 (13.8)	3 (5.8)	11 (10.0)
Chronic obstructive pulmonary disease	6 (10.3)	3 (5.8)	9 (8.2)
Bronchitis chronic	3 (5.2)	0	3 (2.7)
Systemic lupus erythematosus	3 (5.2)	0	3 (2.7)
Pemphigus	2 (3.4)	1 (1.9)	3 (2.7)
Ankylosing spondylitis	1 (1.7)	2 (3.8)	3 (2.7)
Psoriatic arthropathy	0	3 (5.8)	3 (2.7)
Behçet's syndrome	1 (1.7)	1 (1.9)	2 (1.8)

*n* = number of patients with available data; Q1 = lower quartile; Q3 = upper quartile.

a Disorders reported more than once overall; the following disorders were reported once: ulcerative colitis, autoimmune hepatitis, sarcoidosis, giardiasis, osteoarthritis, polymyalgia rheumatica, myasthenia gravis, allergic alveolitis, idiopathic pulmonary fibrosis, pulmonary fibrosis, and psoriasis.

### Efficacy findings

#### QCT analysis

**Fig. 3:**
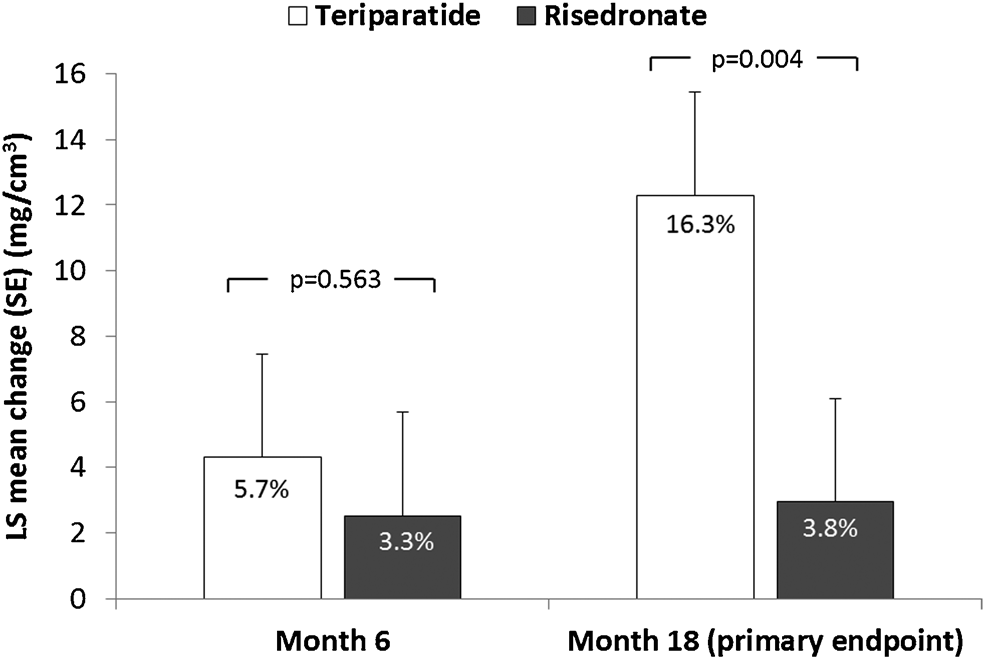
Treatment associated changes from baseline for teriparatide compared to risedronate in lumbar spine (L_1_–L_3_) trabecular BMD measured by QCT. BMD = bone mineral density; LS = least square; SE = standard error. Percentages reflect the percent change from baseline; statistics from a mixed-model repeated measures analysis adjusted for predefined variables (full model with nonmissing data for *n* = 35 and *n* = 37 at month 6, and *n* = 36 and *n* = 34 at month 18 for teriparatide and risedronate, respectively).

At 18 months, patients in the teriparatide group had a significantly greater increase in LS mean (± SE) trabecular BMD at L_1_–L_3_ from baseline than patients in the risedronate group (12.3 ± 3.2 mg/cm^3^ versus 2.9 ± 3.1 mg/cm^3^; *p* = 0.004) (Fig. 3). This corresponds to increases of 16.3% ± 4.2% in the teriparatide group compared to 3.8% ± 4.1% in the risedronate group (Fig. 3). The between-treatment differences at 6 months were not significantly different. Results were confirmed in the analysis based on the reduced MMRM model. The increases from baseline in trabecular BMD were statistically significant for the two treatment groups in the LOCF analysis.

#### HRQCT analysis

**Fig. 4:**
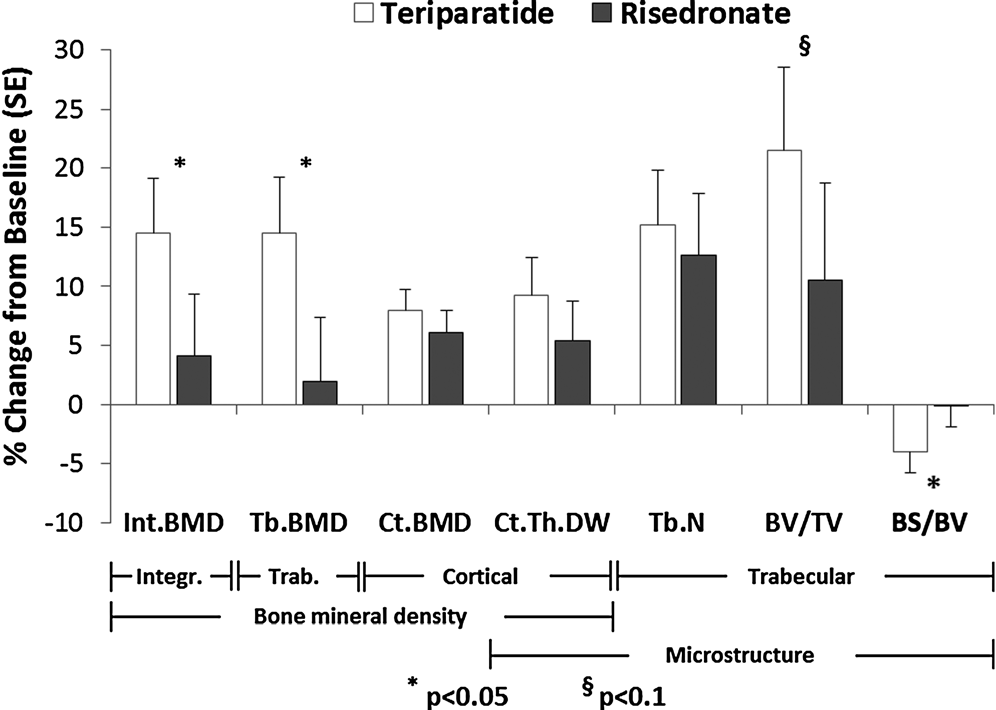
Treatment associated percent changes from baseline to 18 months (LS mean + SE) for teriparatide compared to risedronate in T_12_ BMD, bone microstructure, and one variable affected by density and microstructure (density-weighted cortical thickness), all measured by HRQCT. BMD = bone mineral density; BS/BV = bone surface to volume ratio; BV/TV = bone volume fraction; Ct.BMD = cortical BMD; Ct.Th.DW = density weighted cortical thickness; HRQCT = high resolution quantitative computed tomography; Int.BMD = integral BMD; Integr. = integral; T12 = 12th thoracic vertebra; LS = least square; SE = standard error; Tb.BMD = trabecular BMD; Tb.N = trabecular number; Trab. = trabecular. Percent change from baseline from a mixed-model repeated measures analysis adjusted for predefined variables (full model with nonmissing data for *n* = 28 and *n* = 30 at month 18 for teriparatide and risedronate, respectively). For BS/BV more negative values represent improvement.

**Fig. 5:**
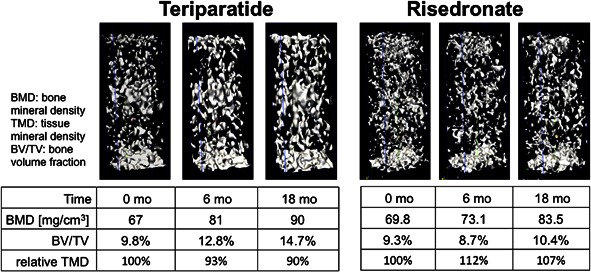
3D visualization of treatment effect for 2 representative patients. BMD = bone mineral density; BV/TV = bone volume fraction; TMD = tissue mineral density. Treatment with teriparatide resulted in a visible increase in bone volume, whereas under risedronate maintenance of bone structure was observed. Bone mineral density increased for both patients. Tissue mineral density was reduced under teriparatide (reflecting apposition of not yet fully mineralized bone) whereas it increased under risedronate due to reduction of bone turnover.

HRQCT results with nonmissing covariates data were available on a subset of 58 patients (28 on teriparatide, 30 on risedronate). Of these, 51 (23 on teriparatide, 28 on risedronate) had nonmissing data at 6 months and 56 subjects (28 on risedronate, 28 on teriparatide) at 18 months (Fig. 4). HRQCT results from the full MMRM model for absolute changes from baseline are summarized in Table 3, and percent changes are depicted in Fig. 4 for a subset of HRQCT variables. Mean changes from baseline to 18 months were statistically significant for all HRQCT variables for both treatment groups, with the exception of the change in the cross-sectional area of the vertebra in the risedronate group. Similar results were observed for the LOCF analysis (data not shown). Examples of the treatment effects for teriparatide and risedronate are visualized in Fig. 5.

**Table 3:** Changes From Baseline to Month 18 in High-Resolution Quantitative Computed Tomography of T_12_

Time point	Teriparatide (*n* = 28)			Risedronate (*n* = 30)			Treatment difference (*p*)
	
	Baseline	LS mean change	SE	Baseline	LS mean change	SE	
Integral BMD (mg/cm^3^)	105.0	15.24	4.86	102.0	4.16	5.34	**0.028**
Trabecular bone BMD (mg/cm^3^)	87.3	12.62	4.14	83.7	1.64	4.57	**0.011**
BMD of cortical VOI (mg/cm^3^)	287	22.89	5.18	291	17.73	5.54	0.328
Apparent BS/BV (mm)	7.75	–0.31	0.14	8.08	–0.00	0.15	**0.032**
Apparent BV/TV	0.13	0.029	0.009	0.12	0.013	0.010	0.098
Apparent trabecular number per area (1/mm)	0.47	0.07	0.02	0.44	0.06	0.02	0.509
Apparent trabecular separation (mm)	4.25	–0.83	0.25	3.93	–0.65	0.26	0.500
Cortical thickness (mm)	2.06	–0.14	0.03	2.01	–0.17	0.03	0.270
Cortical thickness weighted by BMD (mm)	0.25	0.02	0.01	0.25	0.01	0.01	0.240
Cross-sectional area, central slice (mm^2^)	1154	9.82	3.87	1140	7.07	4.17	0.507

Significance of treatment differences derived from the full mixed-model repeated measures analysis adjusted for predefined variables. Bold indicates significant values of *p*.

*n* = number of patients; T_12_ = 12th thoracic vertebra; LS = least squares; BMD = bone mineral density; VOI = volume of interest; BS = bone surface; TV = tissue volume; BV = bone volume.

HRQCT-based increases in T_12_ integral and trabecular BMD were significantly larger for teriparatide compared to risedronate at 18 months (Table 3, Fig. 4). Among microstructural variables, a statistically significant difference was observed for app.BS/BV (*p* = 0.032) and app.BV/TV showed a trend (*p* = 0.098), both in favor of teriparatide (Table 3, Fig. 4). Similar results were observed in the analysis based on the reduced MMRM model where the improvements of both, app.BV/TV and app.BS/BV, were significantly larger for teriparatide compared to risedronate (*p* = 0.045 for both variables). No significant differences between treatments were observed in HRQCT variables at 6 months. All aforementioned HRQCT results of the trabecular region were evaluated in the larger VOI encompassing almost all trabecular bone (Fig. 1*G*, *H*). Results from the elliptical subregion (Fig. 1*F*) were similar (data not shown).

#### FE analysis

**Fig. 6:**
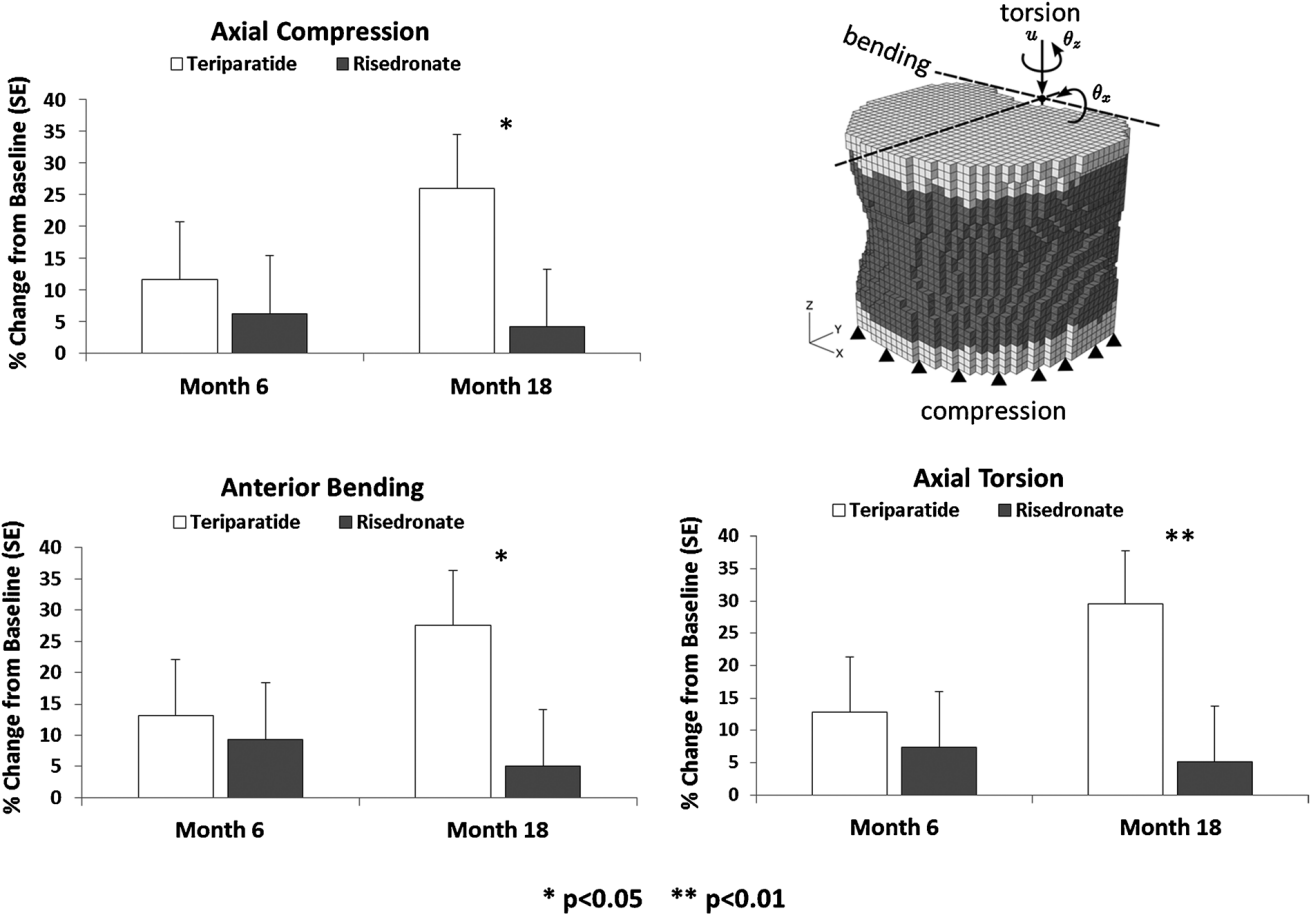
Treatment associated percent changes from baseline (LS mean + SE) in vertebral strength of T12 for teriparatide compared to risedronate as modeled by finite element analysis based on high-resolution quantitative computed tomography; results for three loading modes (top right). LS = least square; SE = standard error; T12 = 12th thoracic vertebra. Statistics from a mixed-model repeated measures analysis adjusted for predefined variables (full model with nonmissing data for *n* = 23 and *n* = 28 at month 6, and *n* = 28 and *n* = 28 at month 18 for teriparatide and risedronate, respectively). Within groups, the increases from baseline in the LOCF analysis were statistically significant for the two treatment groups (*p* < 0.001).

At 18 months, statistically significant increases in vertebral strength were observed for both treatment groups and all three loading modes, with statistically significant larger increases in the teriparatide group (Fig. 6). Similar results were observed for vertebral stiffness, as well as in the LOCF analyses of strength and stiffness (data not shown). Normalized strength in axial compression yielded similar results compared to the non-normalized strength analysis in the teriparatide-treated subjects (26.2% and 26.0% respectively), whereas it showed slightly higher values in the risedronate group (5.8% and 4.2%, respectively), the difference between treatments remained significant (*p* = 0.021). Between-treatment differences were not statistically significant at month 6. Results from the reduced MMRM model were comparable with those from the full MMRM model.

#### Biochemical markers of bone turnover

**Fig. 7:**
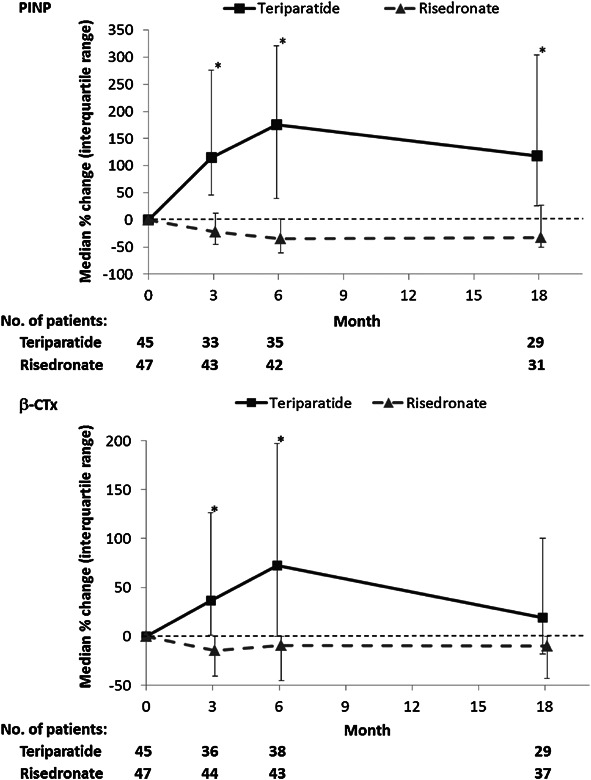
Temporal changes in bone formation (P1NP) and bone resorption (β-CTx) markers; *p < 0.001 for the between-treatment comparison based on mixed-model repeated measures analysis adjusted for selected variables (full model).

The course over time of median percentage changes from baseline for biochemical markers of bone turnover (P1NP and β-CTx) are depicted in Fig. 7. Differences between treatments in the change from baseline were statistically significant at all time points (*p* < 0.001) with the exception of β-CTx at month 18 (*p* = 0.105). As expected, in the teriparatide group, P1NP and β-CTx were increased at 3 months and peaked at 6 months (median increase from baseline of 175.7% [P1NP] and 72.2% [β-CTx]). In the risedronate group, these markers decreased at 3 months and remained suppressed at 18 months (Fig. 7). Between-treatment test results from the reduced model were similar to those from the full model.

#### DXA analysis

**Fig. 8:**
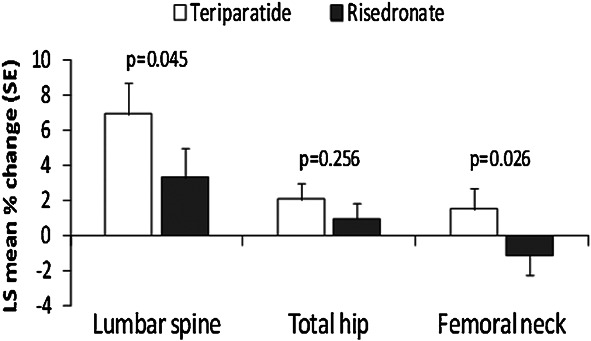
Percent changes in areal BMD measured by DXA between baseline and month 18. BMD = bone mineral density; LS = least square; SE = standard error. Note: *p* values from a mixed-model repeated measures analysis adjusted for selected variables (full model).

After 18 months of treatment, aBMD at the lumbar spine and the total hip significantly increased from baseline for both groups (*p* < 0.05). Changes were statistically significantly higher for teriparatide at the lumbar spine (LS mean ± SE: 0.060 ± 0.015 g/cm^2^ for teriparatide [+6.94%] versus 0.030 ± 0.015 g/cm^2^ for risedronate [+3.33%]; *p* = 0.045), and at the femoral neck (0.011 ± 0.009 g/cm^2^ for teriparatide [+1.52%] versus −0.009 ± 0.009 g/cm^2^ for risedronate [–1.10%]; *p* = 0.026) (Fig. 8). However, between group differences were not significant at the total hip (0.017 ± 0.008 g/cm^2^ for teriparatide [+2.07%] versus 0.008 ± 0.008 g/cm^2^ for risedronate [+0.99%]; *p* = 0.256).

For the primary and secondary efficacy endpoints, results based on the per protocol population, which excluded 16 patients (9 in the teriparatide and 7 in the risedronate group) because of major predefined protocol violations, were comparable to those from the full analysis data sets. Similarly, a post hoc analysis that included the underlying disorder category as a covariate in the fully adjusted model showed almost identical results to the predefined model.

### Safety and tolerability findings

In the teriparatide group, the median (lower quartile [Q1], upper quartile [Q3]) duration of GC therapy during the study was 17.8 months (16.1, 18.0 months), and the median (Q1, Q3) cumulative GC dose was 4.1 g (2.7, 5.6 g). Patients in the risedronate group took GCs for a median (Q1, Q3) duration of 17.5 months (8.9, 18.0 months; *p* = 0.341 compared to teriparatide), with a median (Q1, Q3) cumulative GC dose of 3.1 g (2.4, 5.9 g; *p* = 0.376).

Fewer patients in the teriparatide group than in the risedronate group reported TEAEs or SAEs (Table 4); however, between-treatment differences were not statistically significant (*p* = 0.080 and *p* = 0.089, respectively). None of the patients in the teriparatide group compared to 5 patients (10.6%) in the risedronate group had a new clinical fracture during the study (*p* = 0.056). The 5 patients in the risedronate group had a total of 11 clinical fractures: eight rib fractures and one fracture each at the hip, radius, and ankle (*p* < 0.001 between-treatment analysis from Poisson regression). No clinical vertebral fractures were reported during the study. No cases of hypercalcemia were reported and no clinically relevant findings were seen in the assessment of vital signs, height, weight, and BMI.

**Table 4:** Summary of TEAEs

Preferred term	Number (%) of patients		*p*^a^

	Teriparatide (*n* = 45)	Risedronate (*n* = 47)	
Number of patients with ≥1 TEAE	25 (55.6)	35 (74.5)	0.080
Reported in >4% of patients overall			
Arthralgia	4 (8.9)	3 (6.4)	
Influenza	4 (8.9)	3 (6.4)	
Edema peripheral	3 (6.7)	2 (4.3)	
Chronic obstructive pulmonary disease (exacerbation)	2 (4.4)	4 (8.5)	
Nasopharyngitis	2 (4.4)	2 (4.3)	
Dyspnea	1 (2.2)	3 (6.4)	
Fall	1 (2.2)	3 (6.4)	
Nausea	1 (2.2)	3 (6.4)	
Weight increased	1 (2.2)	3 (6.4)	
Number of patients with ≥1 SAE	13 (28.9)	22 (46.8)	0.089
Reported in >2% of patients overall:			
Intervertebral disc protrusion	3 (6.7)	0	
Chronic obstructive pulmonary disease	2 (4.4)	3 (6.4)	
Crohn's disease (exacerbation)	0	2 (4.3)	
Fall	0	2 (4.3)	
Hypertensive crisis	0	2 (4.3)	
Death	2 (4.4)	1 (2.1)	0.613
Number of discontinuations due to TEAE	0	3 (6.4)	0.242

TEAE = treatment-emergent adverse event; SAE = serious adverse event.

a *p* value from Fisher's exact test.

## Discussion

GIO can have devastating sequels with high rates of morbidity and mortality after a fracture, and significant cost to society with respect to hospital expenses and loss of independence from related complications, in the context of an already debilitating underlying disorder.^28^

In this active-comparator trial of men with GIO, daily subcutaneous teriparatide was more efficacious than weekly oral risedronate to increase BMD at the lumbar spine measured by QCT at 18 months of treatment, which was the maximum treatment duration approved in the participating countries at the time the study was conducted. Moreover, teriparatide-treated patients appeared to show additional significant skeletal benefits compared to risedronate-treated patients, with greater improvements in bone strength and stiffness, as calculated by HRQCT-based FE analysis at the 12th thoracic vertebra. Such improvements were shown for strength under compressive and bending loads, as well as under axial torsion. HRQCT analysis revealed greater increases in integral and trabecular BMD with teriparatide, without significant differences in cortical BMD between the two treatment groups. With the only exception of cross-sectional area of the vertebral body, all other structural variables derived from HRQCT analysis showed statistically significant increases from baseline for both treatment groups. For all microstructural variables, the improvement was somewhat larger for teriparatide compared to risedronate, but only for BS/BV the between-treatment difference reached statistical significance. In the full model, bone volume fraction (BV/TV) showed a trend for greater increase in the teriparatide group (*p* = 0.098), and was statistically significant in the reduced MMRM model (*p* = 0.045).

The assessment of the bone structure and BMD by volumetric QCT and HRQCT at axial fracture sites, such as the spine, with improved spatial resolution and faster scan acquisition empowered by multidetector technology and spiral scanning, allows a differentiated analysis of the different bone compartments, and more accurately reflects the skeletal effects of bone active drugs than conventional DXA. DXA has the disadvantage of being based on a 2D assessment of a 3D structure with resulting bias of the measured aBMD caused by degenerative disorders. 3D imaging modalities provide a better estimation of bone strength at the spine level using FE analysis,^23^,^25^ as well as better prediction of vertebral fracture risk in men.^29^

The results of this trial in men with GIO are similar to previous results in postmenopausal women with osteoporosis where trabecular BMD at the spine, measured with the same QCT software, showed an increase of 19.0% and 3.8% after 18 months of teriparatide and daily alendronate, respectively.^30^ Similar QCT-based responses to teriparatide have been reported from other studies in postmenopausal women.^14^,^31^ Of note, in a study in postmenopausal women with severe GIO who received therapy with a daily teriparatide dose of 25 µg combined with hormone replacement therapy for 12 months, the BMD increase at L_1_–L_2_ was substantially higher (35%).^32^ Similarly, in a 2-year comparator trial in men with non-GC–associated low bone mass, treatment with teriparatide (37 µg/d) increased trabecular BMD of L_1_–L_4_ by 48% compared with 3% in the alendronate daily group.^18^ However, it should be noted that these two latter studies used older QCT software, and therapy was with higher doses of synthetic, non-recombinant teriparatide than in the more recent studies.

This is the first study in which HRQCT was used to study not only bone forming^7^,^14^ but also antiresorptive treatment. A post hoc sensitivity test comparing QCT and HRQCT on the largest common dataset (*n* = 55 at 18 months) confirmed that the discriminatory power of HRQCT for differentiating treatment effects based on trabecular BMD was at least as large as that of QCT (*p* < 0.01 versus *p* < 0.05, respectively).

The higher spatial resolution of HRQCT permits more detailed insight into compartment-specific changes in microstructure, but for interpretation of results, the limits of resolution, ie, partial volume effects, need to be considered.^21^ Teriparatide can be expected to lead to increases in BV/TV, but since the newly added bone matrix is not yet fully mineralized, the magnitude of increases may be underestimated as a consequence of partial volume effects. For risedronate the opposite effect can be expected: increases in TMD may lead to a virtual increase in BV/TV. As a consequence, the difference in treatment effects on BV/TV will be underestimated. Second, although BMD data were cross-calibrated across centers, no such procedure has been implemented yet for measurements of microstructure. Differences between scanners and reconstruction kernels can be substantial, and development of cross-calibration procedures under way should improve microstructural assessment in a multicenter setting. Despite these conditions and the smaller sample size of HRQCT data, we were able to differentiate structural treatment effects based on app.BS/BV. Apparently, the partial volume–related bias of app.Tb.N and app.BV/TV largely cancelled out for app.BS/BV, which was calculated from the ratio of these two measures. As would be expected for an antiresorptive agent, no changes were observed for app.BS/BV for risedronate, whereas app.BS/BV was reduced (ie, improved) for teriparatide, in line with the expected effect of bone apposition. Between-treatment differences in BV/TV were borderline significant, depending on the MMRM model (full or reduced) selected. One should note that this was achieved despite the fact that BV/TV was measured directly as a true microstructural measure, and was not simply derived from BMD as is done with the Xtreme-CT device for peripheral HRQCT.^33^ The latter, by definition, cannot yield independent structural information.

Given the limited spatial resolution, for cortical bone structure we considered it to be difficult to separate treatment effects on thickness from effects on TMD. To maximize sensitivity of a cortical measure, we derived Ct.Th.DW, which reflects the combined effect on both thickness and density. As a consequence, we succeeded in picking up treatment effects for both treatments but at the expense of being unable to differentiate bone forming effects of endosteal apposition (for teriparatide) from increases in TMD (for risedronate). Modifications of cortical measures are under development.

QCT-based FE analysis incorporates vertebral geometry, BMD distribution, and impacting loads to estimate vertebral strength. Microstructural changes such as trabecular thinning, lower BV/TV, and reduced connectivity have been reported for GIO based on histological data^34^–^36^ or micro-CT analysis.^36^ However, to date, it has not been possible to measure these aspects noninvasively in humans.^9^ The publication by Ito and colleagues^6^ confirmed the potential of QCT approaches for assessing vertebral microstructure in vivo. Our HRQCT-based FE results document that there is a stronger effect of teriparatide compared to risedronate on the biomechanical properties of the vertebra in men with GIO after 18 months of treatment. The FE methodology used in this study has been validated by showing that it provides predictions of vertebral maximum load that correlate well with ex vivo human vertebral sections tested mechanically in axial compression.^23^ In this study, we observed highly consistent results for axial compression, anterior bending, and axial torsion, the latter being investigated in a clinical study for the first time. Of note, the improvements in vertebral strength were similar regardless of the loading mode for both drugs. Similarly, the axial compression strength analysis normalized by the vertebral body cross-sectional area did not show major differences with the non-normalized measurement. FE methods could be further refined by finer meshing and incorporation of microstructural data from HRQCT.

In general, the strength results observed in this study were very similar to the FE analysis carried out in postmenopausal osteoporosis in the teriparatide and alendronate comparator trial,^13^ and the OPTAMISE study.^16^ As it was shown in postmenopausal women treated with alendronate,^13^ the biomechanical response to risedronate stagnates after 6 months of therapy, whereas anabolic treatment leads to a further improvement with the additional 12 months of therapy. In fact, extended treatment durations with teriparatide to up to 24 months are associated with substantial increases in the maximum load at the spine of postmenopausal women with severe osteoporosis who had previously been treated with antiresorptive drugs.^14^

Based on the observed differences in BMD and the observed biomechanical responses, teriparatide treatment might be expected to provide better fracture risk reduction benefits in men with GIO. However, this could not be ascertained as this study was not primarily designed to compare incident fracture differences. A trend (*p* = 0.056) in the number of patients with new clinical fractures was observed in favor of teriparatide, where no new clinical fractures were reported in the teriparatide group whereas 5 patients in the risedronate group reported a total of 11 nonvertebral fractures. In a recent clinical trial in patients with GIO, Saag and colleagues^37^ showed that significantly fewer subjects had new vertebral fractures in the teriparatide group compared with the alendronate group (0.6% versus 6.1%, respectively), whereas the incidence of nonvertebral fractures was similar in both groups (5.6% versus 3.7%).

The changes in areal DXA and the response of the biochemical markers of bone turnover were expected, with greater increases in lumbar spine and femoral neck aBMD after 18 months in the teriparatide group, which confirms previous results from a subgroup analysis of 74 men with GIO treated with alendronate or teriparatide.^38^ It also confirms previous reports in patients with GIO, where early increases in bone formation and resorption markers in the teriparatide group compared with a reduction of bone turnover with alendronate were shown,^37^–^39^ which reflects the differing mechanisms of action of bone forming and antiresorptive drugs.^40^ There were no unexpected or disconcerting safety findings for either treatment, and both drugs were generally well tolerated.

Our study has several limitations. Due to more frequent violations of the HRQCT scan protocol, the number of valid postbaseline HRQCT evaluations was smaller compared with the QCT analyzed cases, which negatively impacted the statistical power to show differences between the study groups in HRQCT structural variables. A limitation of the FE analysis is that bone tissue properties are assumed to be constant for all patients and over the course of pharmacological treatment.^41^ Treatment modifies the average level TMD, and for GIO it is known that fracture risk at a given level of BMD is higher compared to primary postmenopausal osteoporosis^42^ moreover, the rapid onset of fractures after initiation of treatment and the similarly rapid return to pretreatment levels of fracture risk after termination of treatment^43^ cannot be explained by changes in BMD alone. However, the magnitude of these effects remains unclear and further refinement of HRQCT may help to address this issue. Another limitation of the study is that the duration of treatment was for 18 months only. Longer treatment may offer even more pronounced advantages.^44^,^45^ The imbalance in anti-TNF use between groups at baseline may reflect the distribution of underlying disorders between groups, with subjects in the risedronate group more frequently reporting musculoskeletal and gastrointestinal disorders than subjects in the teriparatide group. However, a post hoc analysis that included the underlying disorder category as a covariate in the fully-adjusted model showed almost identical results to the predefined model.

Our study also has specific strengths. We studied men with GIO, which is a population of osteoporotic patients that have been scarcely evaluated in clinical trials. In contrast to women with GIO, who normally report very high rates of rheumatologic disorders, such as rheumatoid arthritis and polymyalgia rheumatica, men with GIO have a more heterogeneous spectrum of underlying disorders, including a higher prevalence of chronic respiratory disorders and inflammatory bowel diseases. We applied innovative imaging technology, investigating several aspects of bone microstructure beyond BMD. Our study also presents the first analysis of men with GIO using FE analysis. Using HRQCT instead of QCT created more accurate FE models of the human vertebral body. Loading conditions used for assessing vertebral strength include three simulating models, included axial torsion, which more comprehensively spans the range of loading conditions that occur in vertebral fractures in humans.

In summary, in this study of men with GIO, treatment for 18 months with teriparatide—a bone forming drug—was more efficacious than risedronate—a potent antiresorptive—in improving vertebral bone competence with regard to QCT and HRQCT-based trabecular BMD, HRQCT-based integral BMD, FE-derived bone strength, and bone surface-to-volume ratio as a microstructural variable. Both teriparatide and risedronate were generally well tolerated. Additional studies are needed to elucidate the clinical consequences of these results, and the place of advanced radiologic imaging techniques in the assessment of GIO.

## Disclosures

C-CG and PKZ have received honoraria and research support from Eli Lilly & Company. CN has received honoraria from Eli Lilly & Company. AR's contribution was supported by Eli Lilly & Company. FM, BS, and HP are full-time employees of Eli Lilly & Company. All other authors state that they have no conflicts of interest.
